# ATP6AP1‐CDG: Follow‐up and female phenotype

**DOI:** 10.1002/jmd2.12104

**Published:** 2020-04-09

**Authors:** Patryk Lipiński, Dariusz Rokicki, Anna Bogdańska, Justyna Lesiak, Dirk J. Lefeber, Anna Tylki‐Szymańska

**Affiliations:** ^1^ Department of Pediatrics, Nutrition and Metabolic Diseases The Children's Memorial Health Institute Warsaw Poland; ^2^ Department of Biochemistry, Radioimmunology and Experimental Medicine The Children's Memorial Health Institute Warsaw Poland; ^3^ Department of Nephrology, Kidney Transplantation and Hypertension Children's Memorial Health Institute Warsaw Poland; ^4^ Department of Laboratory Medicine, Translational Metabolic Laboratory Radboud University Medical Center Nijmegen The Netherlands

**Keywords:** ATP6AP1 deficiency, congenital disorder of glycosylation, proteinuria

## Abstract

In 2016, 11 male patients were reported with immunodeficiency and hepatic, gastric and (in some) neurological disease due to X‐linked ATP6AP1 deficiency (ATP6AP1‐CDG). In 2018, three other patients were reported with additional features: connective tissue abnormalities, sensorineural hearing loss, hyperopia, glomerular and tubular dysfunction, exocrine pancreatic insufficiency and altered amino acid and lipid metabolism. We here present a follow‐up of three reported siblings showing progression of deafness to total hearing loss, progressive loss of hair up to alopecia, chestnut skin and, at last follow‐up, in some of them proteinuria. Three female carriers showed a normal serum transferrin isoelectrofocusing but in two of them there was a persistent proteinuria.

## 1 INTRODUCTION

Fourteen patients have been reported with the X‐linked ATP6AP1 deficiency (ATP6AP1‐CDG).^1^, ^2^, ^3^ Key features were immunodeficiency and liver involvement.^1^ Some patients also showed neurological involvement, connective tissue involvement, glomerular and tubular dysfunction, sensorineural hearing loss, exocrine pancreatic insufficiency and/or altered amino acid and lipid metabolism.^2^, ^3^

ATP6AP1 as well as ATP6AP2 are accessory subunits of the vacuolar H^+^‐ATPase, mediating the acidification of eukaryotic cell organelles.^4^ We here present a follow‐up of three reported members of one family and symptoms of female carriers from this family.

## 2 PATIENTS

**Figure 1:**
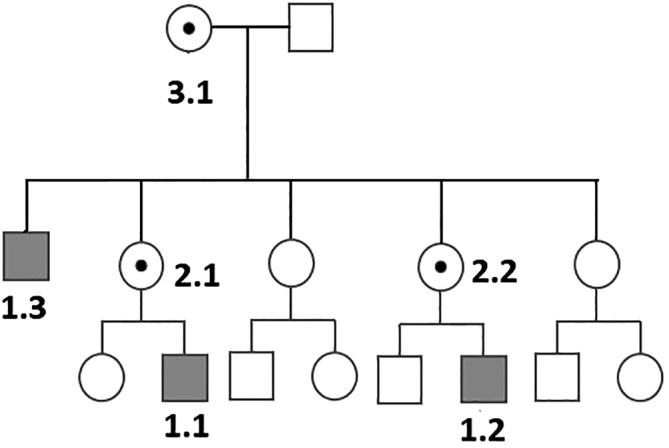
Family pedigree

Three affected males and two female carriers of a multigenerational family were diagnosed and followed at the Children's Memorial Health Institute (Warsaw, Poland). The pedigree of the family is shown in Figure 1.

Patient 1.1 was consulted in our hospital for the first time at the age of 9 years for a sensorineural hearing loss (since 8 years of age), mild liver enlargement with mild elevation of serum transaminases (aspartate aminotransferase (GOT) 70 U/L, alanine aminotransferase (GPT) 76 U/L), first noted at 7 years of age) and normal intellectual development. Birth weight was normal. Bilateral inguinal hernias were observed in infancy. Protein N‐ and O‐hypoglycosylation (increased mono‐, di‐ and tri‐sialotransferrin, increased ApoCIII‐1, decreased ApoCIII‐2) was detected at this consultation. Progressive hair loss from the age of 15 years was observed. At 17 years of age, he presented with normal intellectual development, sensorineural hearing loss requiring cochlear implants, normal liver and spleen volume with mild elevation of serum transaminases (GOT 61 U/L, GPT 55 U/L), dark (chestnut) skin and recurrent plantar abscesses. At the last follow‐up, at the age of 25 years, he was noted to present with total alopecia, mild elevation of serum transaminases (GOT 54 U/L, GPT 46 U/L) and normal results of urine analysis. The patient's phenotype was strikingly similar to that observed in his cousin (1.2) and uncle (1.3).

Patient 1.2 was consulted in our hospital for the first time at the age of 9 years with normal intellectual development, sensorineural hearing loss (since 6 years of age) requiring cochlear implants, anemia and leukopenia (first noted at 4 years of age), hypogammaglobulinemia (IgG 6,1 g/L), normal liver and spleen volume and mild elevation of serum transaminases (GOT 64 U/L, GPT 39 U/L). *Hydrops foetalis* was observed at birth. Bilateral inguinal hernias as well as mild elevation of serum transaminases were present in infancy. Protein N‐ and O‐hypoglycosylation (the same as in his cousin and uncle) was noted at this consultation. At the last follow‐up, at the age of 18 years, he was noted to present with progressive hair loss, persistent leukopenia, normal serum IgG (7,13 g/L), mild elevation of serum transaminases (GOT 46 U/L, GPT 51 U/L) and glomerular proteinuria (2,4 g/24 hours and 76 mg/dL) with normal renal function as well as normal results of renal biopsy.

Patient 1.3 was consulted in our hospital (as part of family screening) for the first time at the age of 30 years with normal intellectual development, normal liver and spleen volume and mild elevation of serum transaminases (GOT 68 U/L, GPT 52 U/L), total alopecia and dark (chestnut) skin. Birth weight was normal. Bilateral inguinal hernias were present in infancy. There was progressive hearing loss from childhood up to total hearing loss in the second decade of life, progressive hair loss since adolescence and mild enlargement of the liver in childhood/adolescence with normal results of liver biopsy. Protein N‐ and O‐hypoglycosylation (the same as in his nephew) was noted at that time. At the last follow‐up, at the age of 36 years, he was noted to present with total alopecia, mild elevation of serum transaminases (GOT 57 U/L, GPT 48 U/L), leukopenia and mild proteinuria (0,89 g/24 hours and 45 mg/dL).

These three patients were hemizygous for the missense variant c.1284G>A, p.M428I in the *ATP6AP1* gene as reported in 2016.^1^

The female carriers (marked as 2.1, 2.2 and 3.1; see Figure 1) showed normal serum protein glycosylation. Two of them (2.2 and 3.1) showed mild proteinuria (0,4 g/24 hours and 0,3 g/24 hours, respectively) at last follow‐up (48 and 75 years of age, respectively). The carrier 3.1 presented also with progressive hearing loss.

## 3 DISCUSSION

The follow‐up of the present patients provides novel insights into ATP6AP1‐CDG showing three patterns of evolution. First, there are symptoms that appear in childhood/adolescence (sensorineural hearing loss, hair loss and hyperpigmentation) and in adolescence(proteinuria). Second, there are symptoms that aggravate with age (from sensorineural hearing loss to total deafness, from hair loss to total alopecia). Third, there is decrease of a symptom (hepatomegaly).A feature not reported in the previous papers on this CDG is proteinuria. This is not unique for CDG; for example, it is a rather frequent finding in Phosphomannomutase deficiency.^5^ Interesting is the presence of proteinuria without other symptoms in some carriers.There is some phenotypic overlap with defects in other vacuolar H^+^‐ATPase subunits: cutis laxa in ATP6V0A2‐CDG, ATP6V1A, ATP6V1E1 and ATP6AP2; liver disease in ATP6AP2‐CDG, CCDC115‐CDG and TMEM199‐CDG; and immunodeficiency in ATP6AP2.^6^, ^7^, ^8^, ^9^, ^10^, ^11^ In males with sensorineural deafness, hair loss and proteinuria, screening for CDG should be performed.
